# Primary renal synovial sarcoma: A case report

**DOI:** 10.1097/MD.0000000000022706

**Published:** 2020-10-16

**Authors:** Bei Zhang, Chao An, Yanjiao Zhang, Junwei Tian, Zhuo Wang, Jiping Wang

**Affiliations:** a Department of Radiology, First Hospital of Jilin University; b Department of Radiology, Third People's Hospital of Shenzhen City, Guang Zhou University of Chinese Medicine, Shenzhen; c Department of Bone and Joint Surgery, First Hospital of Jilin University, Changchun, China.

**Keywords:** case reports, diagnosis, renal neoplasms, synovial sarcoma

## Abstract

**Rationale:**

Synovial sarcoma (SS) is a malignant neoplasm that arises from soft tissues proximal to the joints. It occurs primarily at the major joints of the extremities, but may also occur in the deep soft tissues around the joints. While primary renal synovial sarcoma (PRSS) is extremely rare, it is important to have a better understanding of their imaging and clinical features to establish an effective treatment plan. Correct identification of PRSS is also useful for treating renal neoplasms.

**Patient's concerns:**

A 56-year-old Chinese man was admitted to our hospital due to moderate, paroxysmal left-sided loin pain.

**Diagnosis:**

Renal enhanced computed tomography (CT) scanning showed a relatively hypovascular lesion with calcification in the left kidney. A radical nephrectomy was performed in the left kidney. Postoperative pathology indicated SS with necrosis. The immunohistochemical findings were as follows: 34βE12 (Epithelium+), Bcl-2(+), CD99(+), CK-pan((Epithelium+), EMA(Epithelium+), Ki-67(+60%), and Vimentin(+), CD34(−).

**Interventions:**

The patient underwent radical left nephrectomy with no complications.

**Outcomes:**

After discharge, a close review for 3 months showed no evidence of recurrence.

**Lessons:**

PRSS should be considered for the differential diagnosis of renal hypovascular tumors. When problems arise in distinguishing renal hypovascular tumors, surgical pathology is helpful in the final diagnosis and further treatment of the disease.

## 1 Introduction

Synovial sarcoma (SS) is a rare type of cancer, which occurs in 5% to 10% of soft tissues.^[1]^ It tends to arise the most frequently in the lower limbs of the body. However, tumors may develop in other parts of the body, that is, head, neck, trunk, and pelvis.^[2]^ Primary renal synovial sarcoma (PRSS) is rarer compared with SS.

SS typically occurs in young adults and has a poor prognosis, with a tendency to relapse and metastasize at a late stage.^[3]^ Currently, surgery is the only option for comprehensive treatment. Radiotherapy, chemotherapy, and targeted molecular therapy are considered as additional options following surgery.^[4]^

There are several forms of hypovascular renal tumors. But the conclusive features of their imaging manifestations are missing. When the tumor volume increases and internal degeneration and necrosis develop, the internal components become complicated and often difficult to distinguish by imaging. As a result, a proper understanding of the imaging manifestations of PRSS may prompt clinicians to make an early diagnosis and apply the appropriate treatment.

## 2 Case presentation

The case was a 56-year-old Chinese man with a 1-month soreness in the left waist that worsened in the last 2 days. On May 8, 2018, he was admitted to the hospital, while during hospital admission, the symptoms were not alleviated or treated. The patient had no family history of a malignant tumor. He was the administrator of a financial institution without any history of smoking and alcohol abuse.

**Figure 1:**
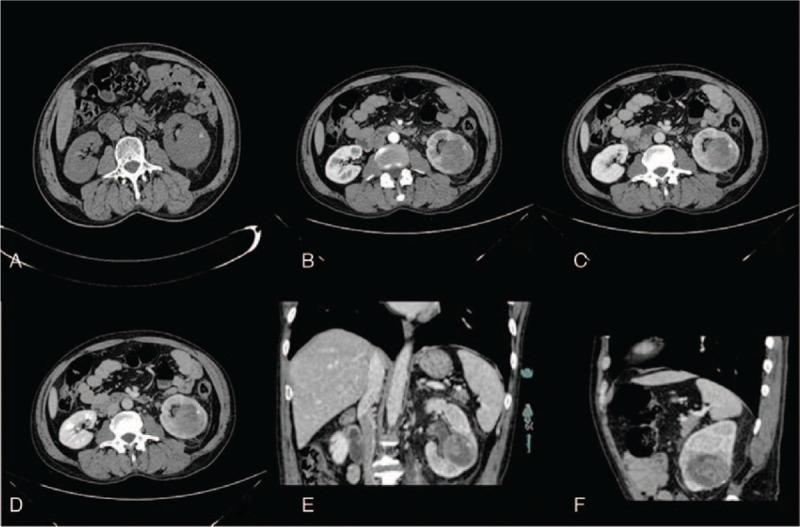
CT imaging. Axial plain CT scan (A) showed space-occupying lesions in the left kidney and about 4.5 cm × 4.0 cm in size. CT value is 25 to 35 Hu with calcification. The boundary of the lesion looks unclear. Axial enhanced CT (Figure B–D) are cortical phase, medullary phase, and excretory phase in order. The enhancement of the lesion was not obvious. A relatively low-density area is visible within the lesion. Coronal and anomalous images of the medullary phase of lesions are displayed in E and F. CT = computed tomography.

Physical examination showed that the angles of bilateral ribs were symmetrical, and there was no protuberance in the bilateral kidney area. There was negative bilateral renal tenderness and discomfort in tapping. The tumor makers, routine urine, blood tests, and other relevant laboratory tests did not show any significant findings. The abdominal computed tomography (CT) scan revealed a circular mixed density of about 4.5 cm in diameter in the left kidney. CT value had a calcification margin of about 34 to 37 HU, shown in Fig. 1A. A CT-enhanced scan revealed a slight enhancement that is lower than that of the renal parenchyma (Fig. 1B–D). However, the distinction between the lesion and fatty tissue of the renal sinus was not clear (Fig. 1E–F). Surgical resection was advised when the diagnosis of a malignant tumor could not be avoided. Laparoscopic radical nephrectomy was performed on May 12, 2018, under general anesthesia.

**Figure 2:**
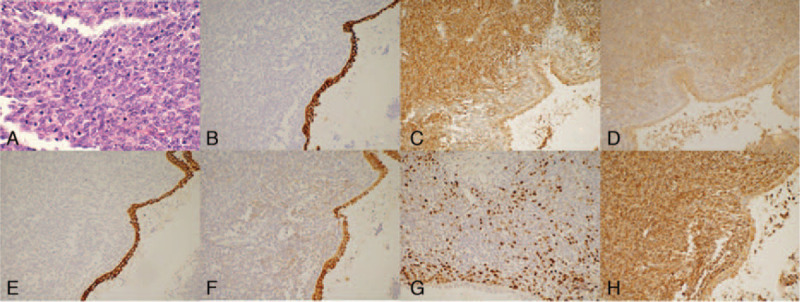
Pathological results. Pathological results (A) indicate that mitosis is clearly seen in mesenchymal differentiation regions (HE, magnification = 400×). Immunohistochemical results (B–H): 34βE12(Epithelium+, 200×), Bcl-2(+, 200×), CD99(+, 200×), CK-pan((Epithelium+, 200×), EMA(Epithelium+, 200×), Ki-67(+60%, 200×), Vimentin(+, 200×).

Postoperative pathology (Fig. 2A) has shown that mitosis was normal in the differentiated mesenchymal areas (haematoxylin and eosin, HE, magnification = 400×). The results of immunohistochemical findings (Fig. 2B–H) were as follows: 34βE12 (Epithelium+, 200×), Bcl-2(+, 200×), CD99 (+, 200×), CK-pan((Epithelium+, 200×), EMA(Epithelium+, 200×), Ki-67(+60%, 200×), and Vimentin(+, 200×). Histopathological findings indicated an ultimate diagnosis of PRSS with necrosis. The patient was discharged 10 days after surgery (May 22, 2018) without any complications. He was advised to go in for further treatment at the Cancer Center. The patient was re-examined 3 months after surgery. An abdominal and pulmonary CT found no recurrence or metastasis. The patient was recommended to do a once-in-3-month review and long-term follow-up.

## 3 Discussion

SS originates from multipotent stem cells that can be differentiated into mesenchymal or epithelial cells. It accounts for 5% to 10% of all soft tissue sarcomas, with no sex preference.^[5]^ It is a type of malignant mesenchymal tumor with a relatively low occurrence that may arise from all anatomical locations. SS has the potential for multidirectional differentiation with adverse prognosis and is mostly occurs in the extremities and major joints, rarely occurring in renal parenchyma.^[6–8]^ In 2017, 12,390 Americans affected by SS, including adults and children.^[9]^

As a result of tumor necrosis, heterogeneous density in PRSS appears, showing an uneven development in the contrast-enhanced scan.^[10]^ Thus, the probability of hemorrhage and necrosis increases with an increase in tumor volume resulting in liquefied necrosis areas in enhanced scanning of the characteristic imaging manifestation of PRSS.^[11]^

PRSS is one of the sarcomas that are most frequently calcified. For about 30% of cases, calcification may.^[12]^ Another significant clinical finding is that PRSS may appear on the CT scan as a mass with calcification. Although the exact reason is still uncertain, these characteristics may be related to its histological subtype. Histologically, SS can be classified into 4 types, namely, biphasic, monophasic spindle-cell type, monophasic epithelial, and poorly differentiated types. The most common forms of calcification are biphasic and monophasic type.^[13]^ Here, the case report of PRSS is a monophasic type combined with calcification. There were no other clinical symptoms in this case except indolent soreness of the left waist.

Hypovascular renal tumors are a group of tumors with identical imaging traits but generally lack the specificity of imaging. However, PRSS should be differentiated from hypovascular clear cell renal cell carcinoma, papillary renal cell carcinoma, and chromophobe cell carcinoma. Some chromophobe cell carcinomas and renal oncocytomas may have the “central stellate scar” sign.^[14]^ Clear cell renal cancer is a blood-rich tumor, but when it is accompanied by hemorrhage and necrosis, it is hard to distinguish from PRSS in terms of development. Nevertheless, on the T2WI sequence of magnetic resonance (MR), the signal intensity of clear cell carcinoma is higher than that of PRSS, chromophobe cell carcinoma, and eosinophilic cell tumor. It helps to differentiate between renal clear cell carcinoma and the others. In the present case, however, the patient did not undergo MR analysis, thereby, further MR imaging features could not include.

Since >90% of SS has t (x; 18) (p11; q11) chromosome translocations, resulting in the fusion of genes SS18-SSX1 or SS18-SSX2,^[15]^ histopathological HE and immunohistochemical staining are very effective in the diagnosis of PRSS.^[16]^ However, a combination of cytogenetic or molecular genetic techniques may improve diagnostic accuracy.

Surgical treatment is still the first choice for synovial sarcoma, including PRSS, if the tumor can be completely removed. Preoperative adjuvant chemoradiotherapy was used for treating SS. However, the effect of preoperative adjuvant chemoradiotherapy is uncertain. Of course, surgery should be performed as soon as SS is detected, considering that PRSS will increase rapidly within a short period. The residual tumor in the margin has been associated with the recurrence of synovial sarcoma. Postoperative chemotherapy is commonly used, but the effect of chemotherapy is not satisfactory, and recurrence was reported within 3 years.^[17]^ Targeted therapy has also been tested and found to be successful.^[18]^

Synovial sarcoma is generally considered to have high malignancy with poor prognosis. Synovial sarcoma is not very responsive to radiotherapy and chemotherapy in both preoperative and postoperative cases. Targeted therapy can be used as an option, but systematic research and data support are still lacking. Therefore, a high rate of local recurrence and metastasis was observed, while data showed that the survival rate was 20% to 50% in the last 5 years.^[7]^

In this case, the CT examination of PRSS was completed, including plain and enhanced scans, which could show the CT imaging features. After the operation, the patients were examined by pathology and immunohistochemistry. It has accumulated valuable information in the imaging and pathological characteristics of PRSS.

Although we reported here an important problem, it also has many limitations. At first, the follow-up time was insufficient for the patient. In view of this, we organized a telephonic follow-up 25 months after the patient was discharged from the hospital (July 5, 2020). The patient said he had received 6 cycles of doxorubicin combined with cyclophosphamide chemotherapy, and during the chemotherapy, there was no progression of the disease and obvious adverse reactions, and the patient was in good condition. Secondly, the patient did not undergo a complete MR examination during these 25 months, which made it impossible to analyze and interpret the first-hand MRI data of PRSS. Besides, the blood samples were not available, resulting in a lack of confirmation at the molecular level.

PRSS has certain imaging characteristics, including solid or cystic, solid tumors, heterogeneous enhancement, proneness to hemorrhage, and necrosis within the tumor, as well as calcification and renal hypovascular tumors. Sometimes, their imaging manifestations lack specificity. Correct and comprehensive understanding of the clinical and imaging manifestations of PRSS is helpful in early diagnosis and timely initiation of a rational treatment protocol.

## Author contributions

**Resources:** Chao An, Yanjiao Zhang, Junwei Tian and Zhuo Wang.

**Writing – original draft:** Bei Zhang.

**Writing – review & editing:** Jiping Wang.
